# Prediction of wear performance in femoral and tibial conformity in patient-specific cruciate-retaining total knee arthroplasty

**DOI:** 10.1186/s13018-020-1548-4

**Published:** 2020-01-22

**Authors:** Yong-Gon Koh, Kyoung-Mi Park, Hwa-Yong Lee, Joon-Hee Park, Kyoung-Tak Kang

**Affiliations:** 1 Joint Reconstruction Center, Department of Orthopaedic Surgery, Yonsei Sarang Hospital, 10 Hyoryeong-ro, Seocho-gu, Seoul 06698 Republic of Korea; 2 Department of Mechanical Engineering, Yonsei University, 50 Yonsei-ro, Seodaemun-gu, Seoul 03722 Republic of Korea; 3 Department of Anesthesiology & Pain Medicine, Hallym University College of Medicine and Kangdong Sacred Heart Hospital, 150 Seongan-ro, Gangdong-gu, Seoul 05355 Republic of Korea

**Keywords:** Total knee arthroplasty, Patient-specific implant, Conformity, Wear, Finite element analysis

## Abstract

**Background:**

Articular surface curvature design is important in tibiofemoral kinematics and the contact mechanics of total knee arthroplasty (TKA). Thus far, the effects of articular surface curvature have not been adequately discussed with respect to conforming, nonconforming, and medial pivot designs in patient-specific TKA. Therefore, this study evaluates the underlying relationship between the articular surface curvature geometry and the wear performance in patient-specific TKA.

**Methods:**

We compare the wear performances between conventional and patient-specific TKA under gait loading conditions using a computational simulation. Patient-specific TKAs investigated in the study are categorized into patient-specific TKA with conforming articular surfaces, medial pivot patient-specific TKA, and bio-mimetic patient-specific TKA with a patient’s own tibial and femoral anatomy. The geometries of the femoral components in patient-specific TKAs are identical.

**Results:**

The anterior-posterior and internal-external kinematics change with respect to different TKA designs. Moreover, the contact pressure and area did not directly affect the wear performance. In particular, conforming patient-specific TKAs exhibit the highest volumetric wear and wear rate. The volumetric wear in a conforming patient-specific TKA is 29% greater than that in a medial pivot patient-specific TKA.

**Conclusion:**

The findings in this study highlight that conformity changes in the femoral and tibial inserts influence the wear performance in patient-specific TKA. Kinematics and contact parameters should be considered to improve wear performance in patient-specific TKA. The conformity modification in the tibiofemoral joint changes the kinematics and contact parameters, and this affects wear performance.

## Introduction

Total knee arthroplasty (TKA) is considered an extremely effective treatment procedure for knee pain associated with degenerative joint disease [1–3]. Several technologies were recently developed to provide better functional outcomes in TKA. Primary TKA is widely known to provide excellent long-term survivorship rates [4, 5], although it is also affected by relatively high dissatisfaction rates [6, 7]. Dissatisfaction is caused by anterior knee pain, mid-flexion instability, reduction in the range of flexion, and the incomplete return of function [8–10].

Changing demographics and higher expectations for TKA increase the demands for effective surgical techniques and implant designs. Knee anatomy studies have indicated that distinct anatomical differences exist with respect to gender and race [11–13]. To address the aforementioned issues, a few studies have proposed an anatomical approach to TKA by using patient-specific or customized prostheses. Patient-specific TKA attempts to preserve a patient’s anatomical geometry. In patient-specific TKA, both the cutting jigs and the implant are particularly designed for the patient. The same preoperative planning that is used for manufacturing jigs is used for manufacturing patient-specific implants with a native femoral intercondylar notch distance, J-curve, condylar offset, anteroposterior and mediolateral width, native tibial bone size, and coverage [14]. The proposed advantages of this type of a system include an optimal implant-bone contact area, reduced micromotion forces, a reduced need for soft tissue balancing, and increasingly normal joint kinematics [13, 15, 16]. However, there is a paucity of extant studies on polyethylene (PE) wear performances in patient-specific or custom TKA.

In a previous study, PE wear was observed to be the main reason for late revisions [17]. As previously mentioned, the femoral component in patient-specific TKA is designed with a J-curve, and the tibial insert focuses on bone size and coverage. The articular geometry in a general patient-specific tibial insert is derived from the femoral component. There is a paucity of extant studies that evaluate the conformity of the surface curvature between the femoral component and tibial insert. We recently studied the changes of the kinematics and wear with respect to different conformities of the articular surface of posterior-stabilized (PS) TKA designs [18]. However, there was difference between the mechanisms for cruciate-retaining and posterior-stabilized TKAs, and there has been no previous study on the wear prediction of different surface conformities for cruciate-retaining PS TKA.

Therefore, this study aims to compare wear performances with respect to the conformity of the articular surfaces between the femoral component and tibial insert in patient-specific TKA. We apply the conformity of conventional cruciate-retaining (CR) TKA to patient-specific TKA. Four different models are developed as follows: conventional CR TKA, patient-specific TKA with a conventional CR TKA conformity, medial pivot patient-specific TKA, and bio-mimetic patient-specific TKA with patient anatomy curvature in the femoral component and tibial insert. We investigated the contact mechanics and wear performance in the aforementioned four different TKA designs using a computational simulation. We hypothesized that patient-specific TKA used in conjunction with a patient’s anatomy provides optimal wear performance.

## Materials and methods

### Design of patient-specific TKA

**Fig. 1:**
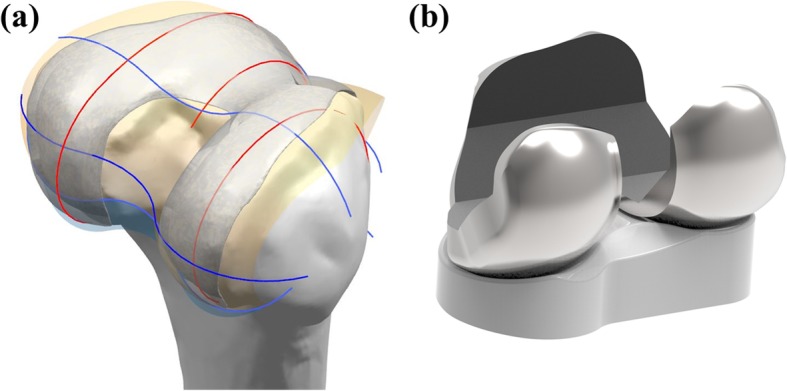
**a** Schematic of patient-specific geometry for the femoral component and **b** developed patient-specific femoral component and tibial insert

The patient-specific TKA is designed using an existing three-dimensional (3D) knee joint model [19, 20]. A patient’s bone is fundamentally used for the patient-specific geometry in the femoral component. The three patient-specific J-curves and the medial and lateral condyles from the patients’ normal articular anatomy are developed using computer-aided design (CAD) software in this study [17, 21–25]. A sagittal plane is introduced in the condyles, in which the anatomy of the articulating surfaces is extracted from the curves. Generally, the femur of a patient in the coronal plane provides asymmetric lateral and medial condyles that are defined as the coronal offset. The aforementioned patient-specific differences are considered in the patient-specific femoral component design (Fig. 1). The coronal offset is defined as the difference in height between the medial and lateral femoral condyles in the coronal plane [17, 26]. It typically supports an asymmetric extension gap between the tibial articular surface and the posterior femoral condyles. The lateral posterior condyle is shorter than the medial condyle and leads to an asymmetric flexion gap [17, 26]. The aforementioned femoral J-curves are matched with patient-specific tibial inserts, and their perimeters correspond to a tibial plateau that restores the distal medial-lateral offset of a patient’s femoral condyles. This is achieved by the height of the patient-specific tibial insert and reflects the condylar offset to maintain normal mechanical axis alignment. The articular geometry in a patient-specific tibial insert used in a previous study was derived from the femoral component [13, 26, 27].

Three tibial insets with different designs are developed. We investigated the articular conformity of conventional conforming CR TKA in the Genesis II Total Knee System (Smith & Nephew Inc., Memphis, TN, USA) and for Evolution Medial Pivot Total Knee Arthroplasty (Wright Medical Technology, Arlington, TN, USA). In order to investigate the conformity of CR TKA, scanning with a non-contact 3D laser scanner (COMET VZ; Steinbichler Optotechnik GmbH, Neubeuern, Germany) with a 50-μm accuracy is used. The scanned point data are converted into 3D models, and scanning is repeated until the 3D model dimensions exhibit geometrical errors of < 100 μm [28].

**Fig. 2:**
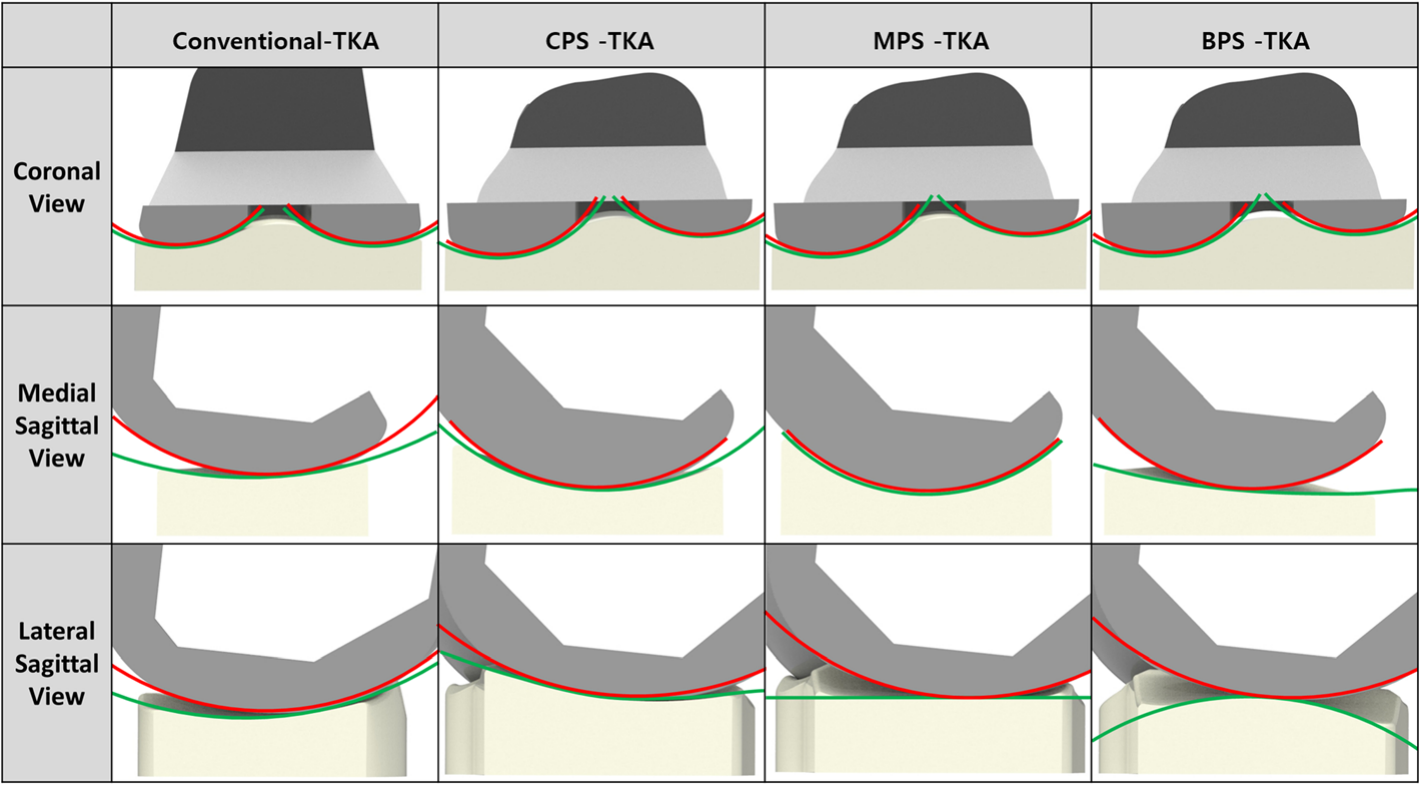
Femoral component and tibial insert designs with different conformities in the conventional and patient-specific TKA

The ratio of the curvature radius for the tibial insert to the curvature radius for the femoral component is investigated for conformity in the coronal and sagittal planes. A tibial insert with a conforming design of conventional CR TKA conformity (Genesis II) and a medial pivot tibial insert with medial pivot conformity (Evolution) are developed by applying the curvature radius ratio in the coronal and sagittal planes to the patient-specific femoral component (Fig. 2). An anatomy tibial insert is developed using a patient’s tibial curvature and is similar to the femoral component. The bone coverage exceeded 95% in the patient-specific TKA, irrespective of differences in the insert design (Fig. 2). All of the femoral component designs in the patient-specific TKA are identical.

### Finite element model

A computational model of the Stanmore knee simulator machine with boundary conditions is constructed in ABAQUS 6.13 (Abaqus, Inc., Providence, RI, USA). An explicit finite element (FE) model based on a study performed by Kang et al. is developed in Abaqus/Explicit [18, 29, 30]. The software program used for modeling and meshing is Hypermesh 11.0 (Altair Engineering, Inc., Troy, MI, USA).

The PE insert is meshed using eight-noded 3D hexahedral elements in four different designs. Femoral and tibial components with high elastic moduli relative to PE (approximately 300 times higher) are developed as rigid bodies that only require surface representation [31, 32]. A convergence test is performed for the optimum mesh density in the tibial insert. Convergence of the analytical solutions for the measurements of the maximum contact stresses within 5% is achieved with the optimum mesh density using elements with a mean edge length of 1.2 mm. Based on a convergence study, the mesh density used for the tibial insert is appropriate [31, 33]. The tibial insert is modeled as an elastic-plastic material with a modulus of elasticity of 685 MPa and a Poisson’s ratio of 0.47 [31, 34]. A penalty-based contact condition is specified at the tibial insert and femoral component interface with a friction coefficient of 0.04 [29, 31].

**Fig. 3:**
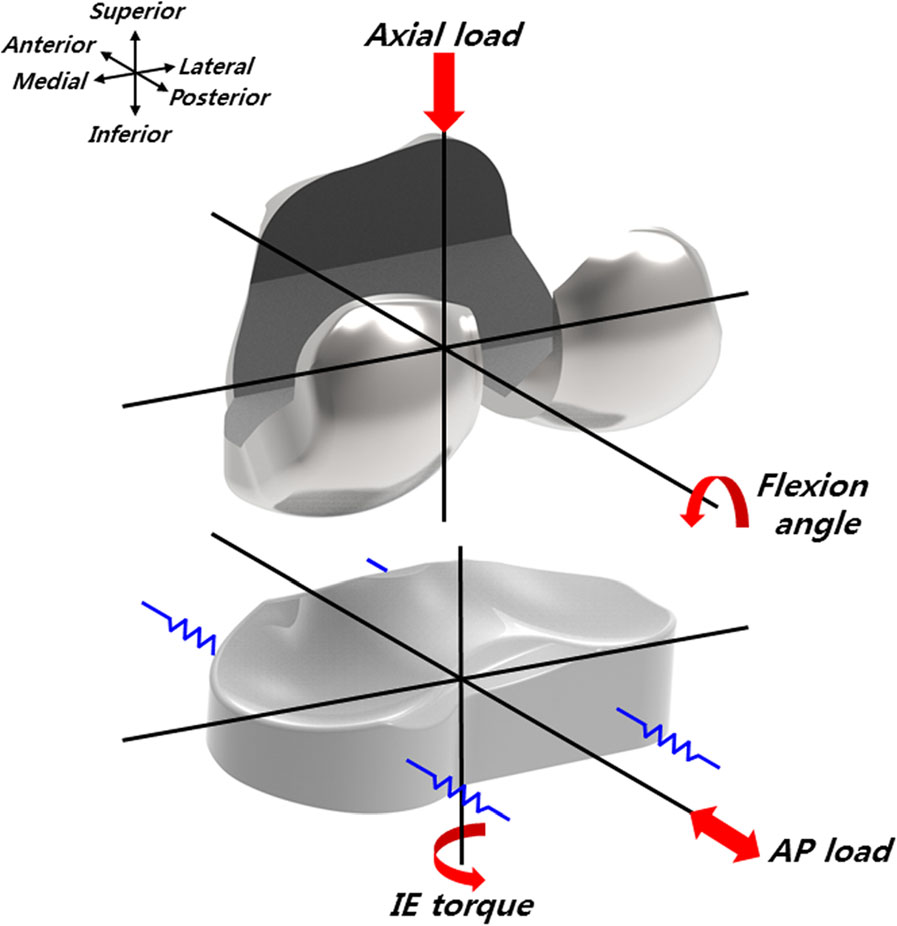
Loading conditions of the FE simulation used in the study

**Fig. 4:**
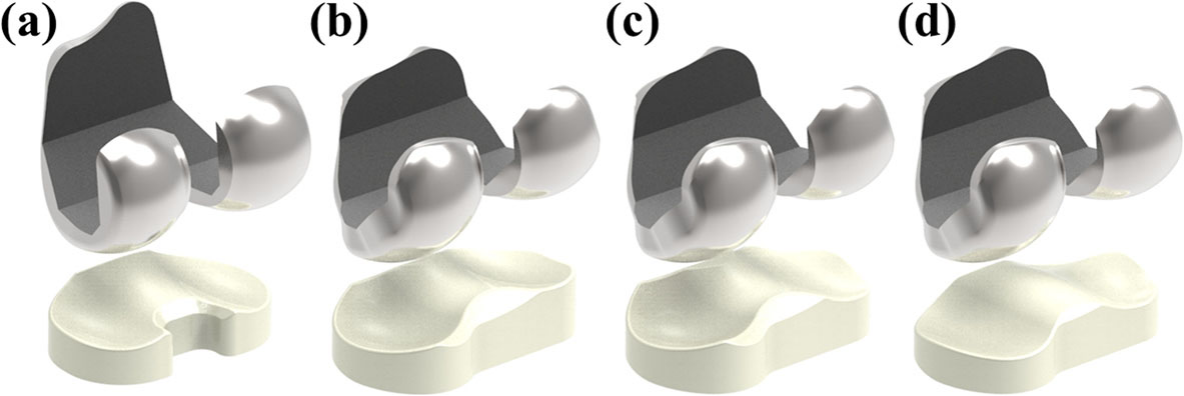
FE models used in this study: **a** conventional TKA, **b** CPS-TKA, **c** MPS-TKA, **d** BPS-TKA

The loading and kinematic conditions obtained from experimental studies are used in the FE simulation (Fig. 3). The model includes simulated soft tissue in the knee simulator with four springs constraining the insert in the anterior-posterior (AP) translation and internal-external (IE) rotation, as well as a spring gap representing anatomical laxity (Fig. 4). The femoral component and tibial insert are the testing conditions with the input profiles of an AP load and IE torque applied to the insert, while a flexion-extension angle and axial force are applied to the femoral component. The axial load, AP translation, and IE rotation are force controlled, and the flexion is displacement controlled. The femoral component is constrained in IE, medial-lateral (ML), and AP, and it is free to translate in the inferior-superior (IS) direction to permit rotation about the frontal and transverse axes to represent varus-valgus (VV) rotations and flexion-extensions, respectively. The axial load application is offset towards the medial condyle to reproduce the 60:40 experimental conditions [31, 33]. The distal surface of the tibial insert is supported in the IS direction, and this is representative of bonded contact with a rigid tibial tray. The tibial insert is allowed to translate in the AP direction and rotate about a fixed vertical axis located in the center of the tibial condyles to simulate IE rotation. The distal surface of the tibial insert is supported in the IS direction, the insert tilt is constrained, and the VV and ML degrees of freedom are unconstrained. A center of rotation for the FE model is directly defined between the medial and lateral condyles. The AP spring translational resistance is 10.4 N/mm, and the IE rotational resistance is 0.30 Nm/deg.

### Wear calculation

The wear process of the PE insert is numerically evaluated by using the Archard wear model [34]. The equation is known as Archard’s wear law (Eq. 1), wherein the linear wear, *H*, is determined as follows:

1 $$ H={K}_{\mathrm{w}} pS $$

Here, *K*_w_ denotes the wear factor, *p* denotes the contact pressure, and *S* denotes the sliding distance. However, the kinematics in total joint replacements are often nonlinear, and thus the applicability of Archard’s law is questionable. Furthermore, in the above expression, delamination, pitting, and third-body wears are not included because previous studies have reported that the aforementioned effects are negligible in PE [35]. In order to include the friction parameter, *μ*, in this model, we adopt the Sakar modification [36] to the Archard model:

2 $$ H={K}_{\mathrm{w}} pS{\left(1+3{\mu}^2\right)}^{0.5} $$

**Fig. 5:**
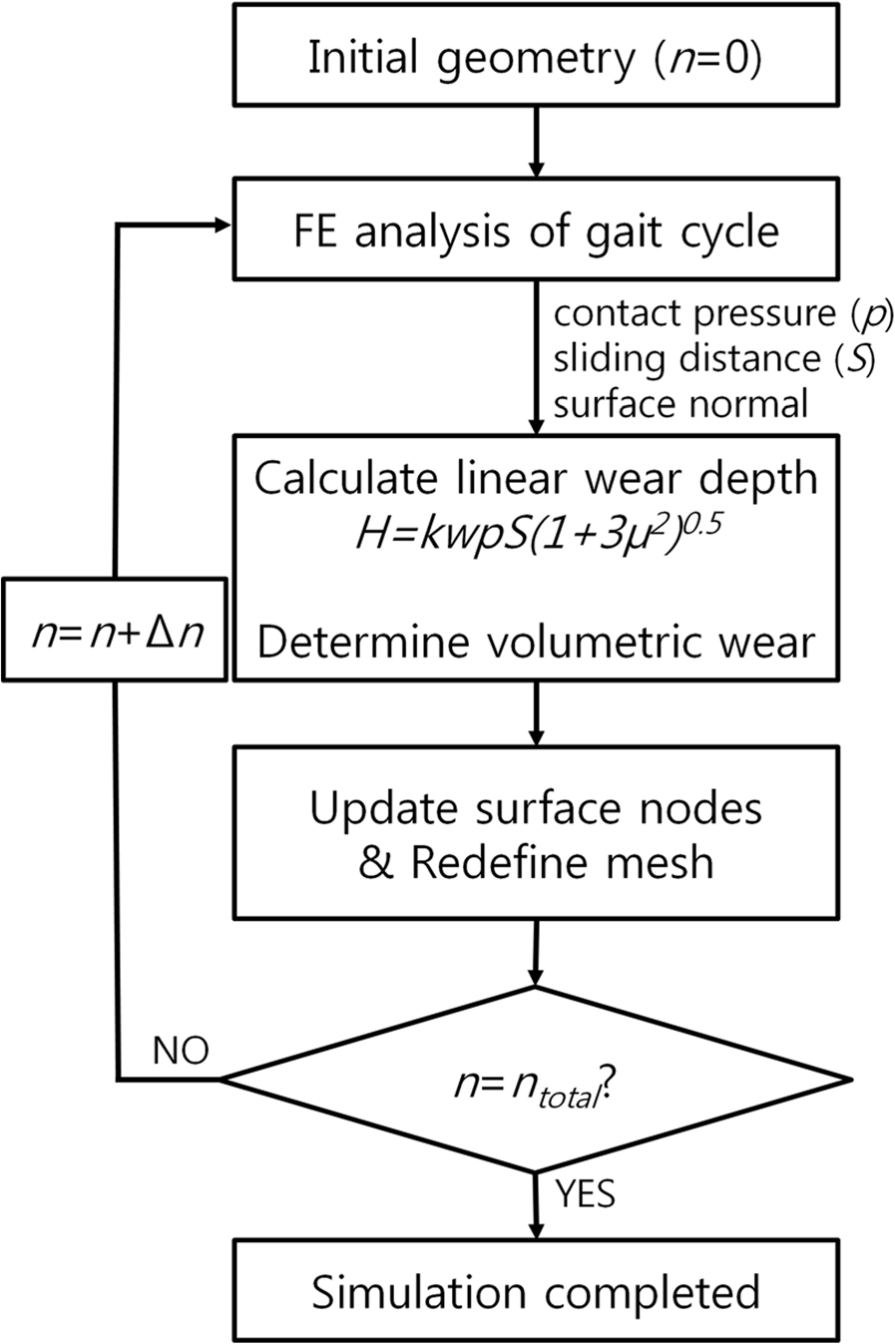
Flowchart of the wear calculation

Each cycle is divided into 100 increments, and wear is computed for each increment and summed during the cycle. The surface nodes influenced by wear move in a direction normal to the articular surface based on the computed material loss at the end of each increment. An adaptive remeshing procedure is introduced to simulate the surface wear progression (Fig. 5). An adaptive wear simulation is performed using Python scripts (Stichting Mathematisch Centrum, Amsterdam, the Netherlands) to interface with the Abaqus output database. The model for the wear calculation of the tibial insert is incorporated into the user subroutine VFRICTION, and this is developed using Fortran. The simulation is iterated and the wear is multiplied by the size of each step (50,000 cycles per step) to evaluate the total wear that occurs during 5 million cycles. The update interval is shorter than those used in previous FE analysis studies on TKA wear [32, 35, 37]. The computed volumetric wear is converted to gravimetric wear using a PE density of 0.93 mm^3^/mg. The wear factor used in this study is estimated using an average of the wear factors from the TKA and ball-on-flat wear tests that were performed in a previous study [38].

We evaluate the wear performances of four different TKAs as follows: conventional Genesis II TKA, patient-specific TKA with Genesis II conformity (CPS-TKA), patient-specific TKA with medial pivot conformity (MPS-TKA), and bio-mimetic patient-specific TKA with a patient’s own tibial and femoral anatomy (BPS-TKA). In order to validate the wear model, the kinematics of conventional TKA are compared with previously obtained experimental data [39]. Additionally, the AP and IE kinematics, contact stresses, contact areas, wear rates, volume metric wears, and wear depths are compared between the four different designs.

## Results

### Wear performance on conformity in patient-specific total knee arthroplasty

**Fig. 6:**
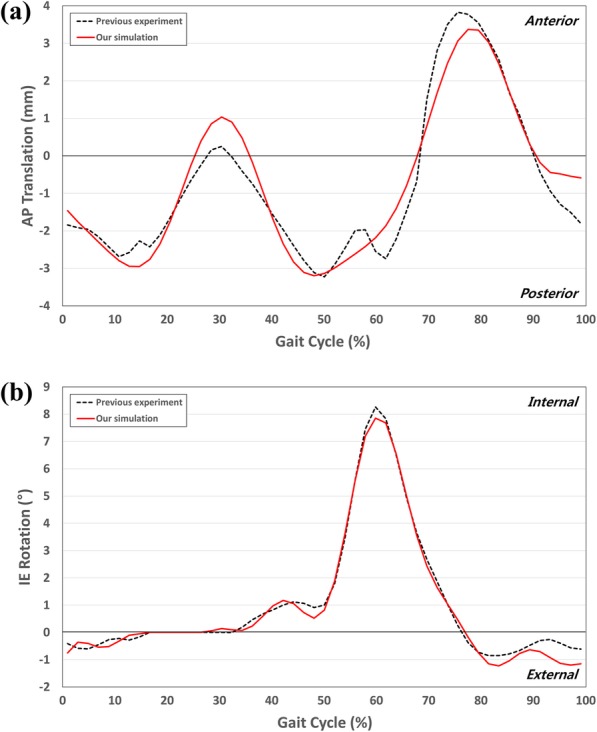
Comparison of **a** AP translation and **b** IE rotation kinematics between the FE model and previous experiments for validation purposes

The conventional TKA model was validated in a previous study [30]. We compared the aforementioned kinematics with data obtained from a previous study, in which experiments were performed under identical loading conditions [39]. The current conventional TKA and validation exhibit good agreement with the predicted and experimental kinematic data [39]. The AP translation and IE rotation are reasonably similar to those of the experimental data in terms of their trends and magnitudes (Fig. 6). The ranges of the AP translation and IE rotation from the experimental data are 7.1 mm and 9.3°, respectively, and the predicted displacement ranges are 6.7 mm and 8.9°, respectively.

### Comparison of the kinematics, wear rates, weight losses, and wear depths between four different designs

**Fig. 7:**
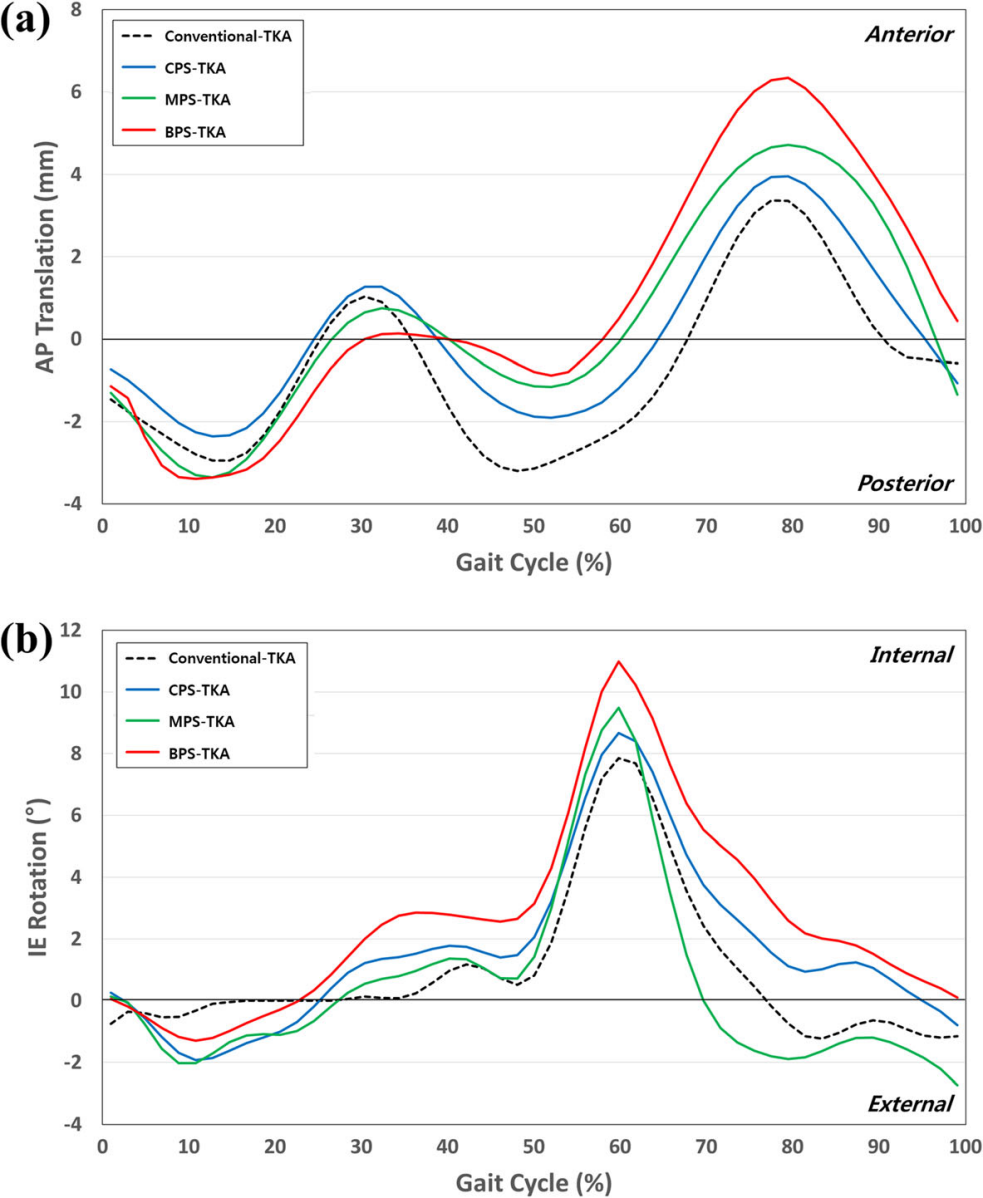
Comparison of **a** AP translation and **b** IE rotation kinematics between the conventional TKA and patient-specific TKA with different conformities

Figure 7 shows the kinematics of the AP translation and IE rotation with respect to the four different designs during a gait-cycle condition. There are differences in the AP translation and IE rotation for each TKA design. Specifically, different kinematics are observed in BPS-TKA. With respect to the AP translation, the anterior translation is observed during the swing phase for the four different designs. However, the anterior translation is 2.9 mm higher during the swing phase in the BPS-TKA when compared to that in the conventional TKA. With respect to the IE rotation, all TKA experienced external and internal rotations during the stance and swing phases, respectively. With respect to the BPS-TKA, a maximum difference of 2.8° is observed during the swing phases when compared to that of the conventional TKA.

**Fig. 8:**
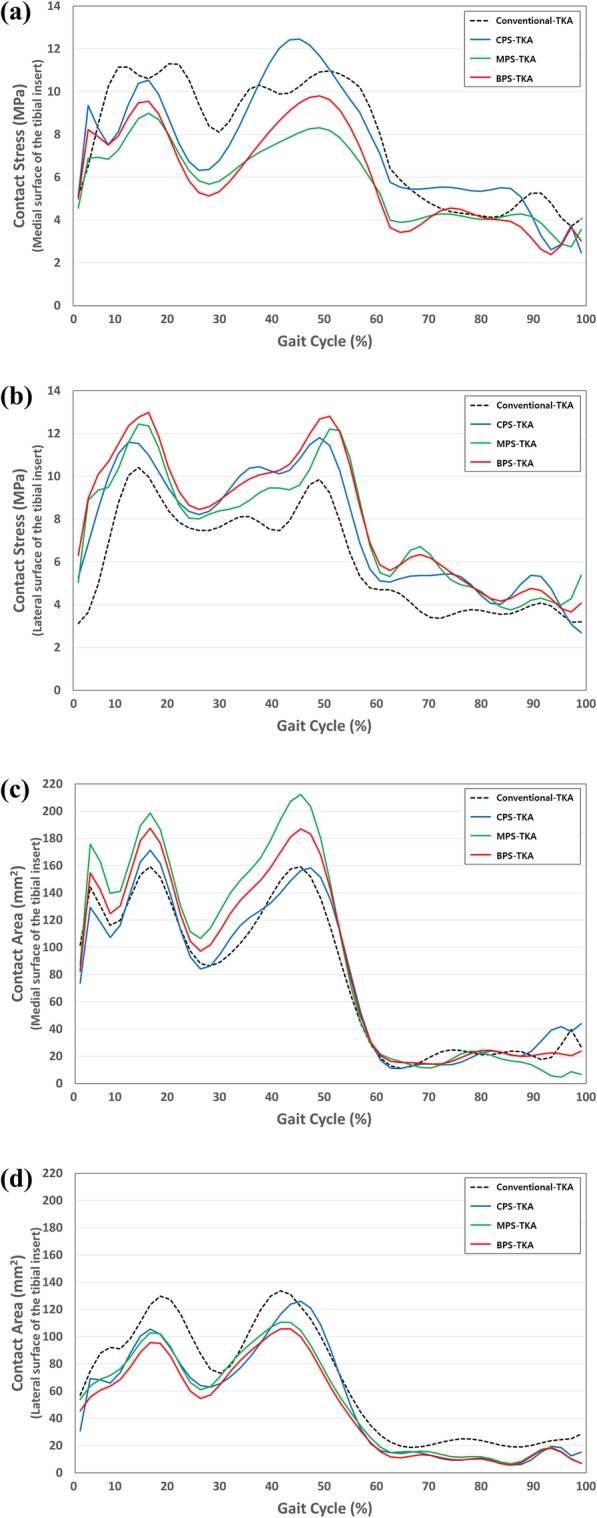
Comparison of the **a**, **b** contact stresses of the tibial inserts and **c**, **d** contact areas of the tibial inserts between conventional TKA and patient-specific TKA with different conformities

Figure 8 shows the contact stresses and areas in the four different designs during the gait cycle. The contact stresses and areas tended to be opposite to each other. This tendency is revealed for all of the different TKA designs. Specifically, the medial pivot design MPS-TKA exhibits a high contact area on the medial side and this leads to low contact stress. However, this pattern is not retained on the lateral side.

### Wear performance on conformity in patient-specific total knee arthroplasty

The predicted wear rates are 21.1, 19.9, 25.7, and 23.2 mm^3^/million for the BPS-TKA, MPS-TKA, CPS-TKA, and conventional TKA, respectively. The predicted volumetric wear is 98.1, 92.5, 119.5, and 116.1 mg for BPS-TKA, MPS-TKA, CPS-TKA, and conventional TKA, respectively, after 5 million cycles. The CPS-TKA exhibited 22%, 29%, and 3% higher volumetric wear when compared to that of the BPS-TKA, MPS-TKA, and conventional TKA, respectively. The maximum wear depths are 0.30, 0.26, 0.35, and 0.31 mm in BPS-TKA, MPS-TKA, CPS-TKA, and conventional TKA, respectively, after 5 million cycles.

**Fig. 9:**
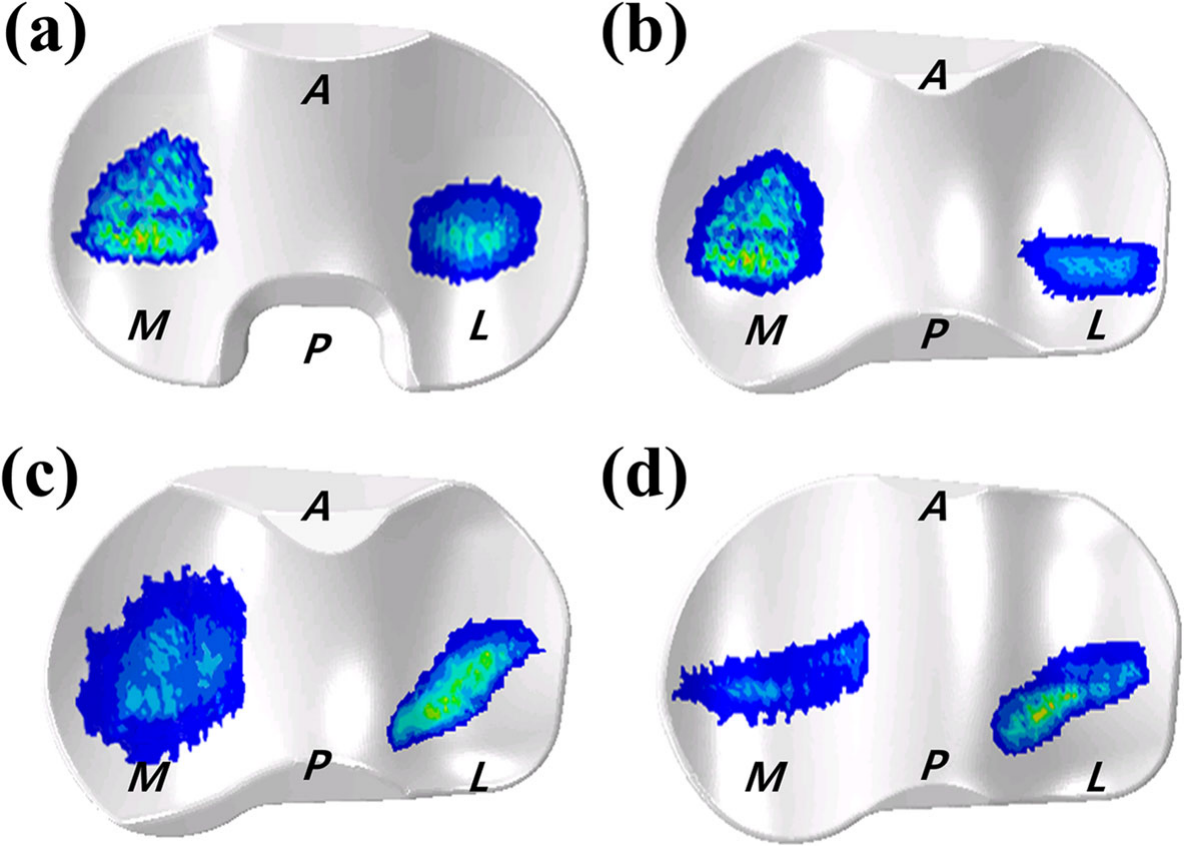
Predicted wear contours of the four different TKA designs: **a** conventional TKA. **b** CPS-TKA, **c** MPS-TKA, **d** BPS-TKA

Figure 9 shows the wear contours for the four different TKA designs. The wear contour exhibits a deep wear region near the center of the medial side and a shallower wear scar on the lateral side with the exception of the BPS-TKA and MPS-TKA. The BPS-TKA and MPS-TKA indicate that the lateral wear region is deeper and wider than the medial side. All data analyzed during this study are included in this manuscript.

## Discussion

The main finding of the present study indicates that mimetic BPS-TKA using a patient’s anatomy geometry does not provide enhanced wear performance, and the proposed study hypothesis is therefore rejected.

The patient-specific approach to TKA is introduced to improve functional outcomes and satisfaction rates [16]. The advantages of the patient-specific system include more normal femoral rollback [13], better coverage over the tibial plateau [15], and fewer incidences of blood transfusions and adverse events [40]. However, there is a paucity of studies that evaluate the wear performance of patient-specific TKA developed using articular surface conformity. Therefore, the aim of this study involves evaluating wear performance, and the current patient-specific TKAs are categorized into three different groups in conjunction with conventional TKA. The patient-specific TKAs are categorized as follows: (1) CPS-TKA with a conforming tibial insert with a corresponding femoral component, (2) MPS-TKA with medial pivot conformity, and (3) BPS-TKA including a bio-mimetic tibial insert with tibia anatomy.

Computational modeling is performed to evaluate wear simulations for four different TKA designs. An increasing number of tribology studies concerning the evaluation of PE wear in TKA have reported the need for improving the understanding of these processes to determine new solutions and avoid the failure of an implant due to PE wear. Experimental studies are often used, although they are neither cost nor time efficient, and they typically analyze only limited configurations and load conditions. Thus, the use of computational modeling is expanding in this research field. Furthermore, measuring wear in vivo after TKA is technically difficult. In this study, a model to predict PE wear is developed using FE analysis.

The results indicate that CPS-TKA provided worse results in terms of wear performance when compared with conventional TKA. However, BPS-TKA and MPS-TKA produced better results in terms of wear performance when compared with conventional TKA. Patient-specific TKA is designed with a femoral component based on a J-curve and optimum bone coverage [21–27, 41]. However, a PE insert can potentially restore the joint-line-corresponding femoral component to improve bony coverage. Thus, the PE insert does not follow a patient’s anatomy.

Furthermore, MPS-TKA with medial pivot conformity exhibits better wear performance. Based on in vivo and in vitro studies of the physiological movements during flexion, the medial femoral condyle does not significantly translate in the AP direction while the lateral condyle moves significantly backward [42–44]. Thus, the medial pivot geometry exhibits a highly conformed “ball-in-socket” design to reproduce the medial pivot motion on the medial side. Several studies have indicated that during flexion (termed as a “medial pivot”), a normal knee provides minimal movement of the medial femoral condyle and a posterior translation of the lateral femoral condyle [45–48]. The implant is introduced to provide the aforementioned characteristics. The medial side exhibits a design with an identical curvature radius on the coronal and sagittal planes to recreate a sphere. The lateral side is smaller than the medial with a cylindrical configuration, and it can stabilize the knee and control rotation. Additionally, the PE asymmetrical insert exhibits a high medial side congruence and a minor lateral congruence. The aforementioned innovations allow for lateral-side posterior sliding and rolling, while the medial side functions as a pivot during knee flexion and guarantees AP stability. The medial pivot design provides better stability and range of motion, less wear stress on the tibial surface, and longer PE survival. These phenomena are reduced by a medial pivot design with better stability and important PE wear reduction [49–55]. Therefore, as previously mentioned, MPS-TKA exhibits better wear performance than CPS-TKA, BPS-TKA, and conventional TKA.

The kinematic data of MPS-TKA were similar to those of BPS-TKA. Interestingly, BPS-TKA that mimicked a patient’s anatomy does not exhibit improved wear performance over MPS-TKA. Patil et al. recently indicated that patient-specific TKA generated kinematics that more closely resemble normal knee kinematics than conventional TKA by using in vitro cadaveric experiments [13]. Additionally, Koh et al. indicated that restoration of the normal geometry of the knee joint in patient-specific bicruciate-retaining TKA and preservation of the anterior cruciate ligament lead to improvements in kinematics when compared with conventional CR and bicruciate-retaining TKA by using computational simulations [27]. Both the aforementioned studies indicate that the patient-specific TKA design of a PE insert conformity is derived from the femoral component.

Generally, TKA knees have exhibited less femoral rollback and reduced internal rotations when compared to normal knees [56]. Interestingly, BPS-TKA has been found to exhibit higher AP translation and IE rotation. However, the wear performance does not reflect the kinematic results. Theoretically, a low conformity exhibits high contact pressure and leads to high wear. However, recent studies have indicated different wear performance trends under different conformities. Abdelgaie et al. indicated that the predicted wear rates for curved inserts (high conformity level) are more than three-times those of flat inserts (the lowest conformity level) [57]. Additionally, Brocket et al. demonstrated a reduction in wear by reducing implant conformity [58]. The study demonstrated that bearing conformity significantly impacts the wear performance of a TKA and provides opportunities to improve clinical performance through enhanced design selection [58]. The TKA design conformity model in this study retains the findings of the aforementioned research and theoretical content. In the native knee anatomy, the medial and lateral tibial plateaus include asymmetric geometries with a slightly dished medial plateau and a convex lateral plateau [59]. Therefore, the BPS-TKA lateral side does not exhibit any conformity because the lateral side is convex. Thus, the BPS-TKA design corresponds to decreased conformity, and the wear performance is better than that of CPS-TKA and conventional TKA. However, the BPS-TKA wear performance is worse than that of MPS-TKA. Based on Archard’s wear law, wear is proportional to the contact pressure, contact area, wear factor, and sliding distance. Therefore, these results are reasonable. A previous study indicated that the increased sliding distance on the lateral side leads to an increased effect on wear when compared with the higher loading on the medial side [60]. The aforementioned studies indicate the reliability of our results. It is interesting to note that the results of the volumetric wear and wear depth are not in agreement for the TKAs based on different designs. Small contact areas with high contact stresses generate higher linear penetration (wear depth) when compared with volumetric wear, while large contact areas with low contact stresses increase the volumetric wear with lower linear penetration (wear depth). This distinction is important since a tendency towards higher linear penetration is more likely to result in localized damage to the insert while a tendency towards higher volumetric wear (with low linear penetration) is more likely to generate a higher volume of wear debris and result in an osteolytic reaction. Therefore, it is possible to select an optimal conformity level to achieve a balance between the kinematics and contact performance.

In terms of clinical relevance, our study indicates that tibial plateau conformity is important when designing patient-specific TKAs. The possibility of setting the conformity based on the patient’s characteristics when designing patient-specific TKAs is also indicated. Additionally, although BPS-TKA does not exhibit the best wear performance, it improves the performance through the optimization process. Wear is the main cause of late revision. Furthermore, patient-specific femoral components follow a normal kinematic trend [13, 27]. Therefore, our results suggest that it is important to carefully determine conformity when a PE insert is designed in patient-specific TKA.

This study includes three limitations. First, our model included a constant wear factor that did not change with the contact stress or sliding direction. Second, it is possible to compare in vitro experimental wear data and measured wear data in a computational simulation, although it is not possible to compare actual clinical wear data. However, the loading condition for 5 million cycles represents a clinical wear situation that is not completely realistic and exhibits limited applicability. Finally, only conventional TKA models are validated using experimental data. However, the advantage of a computational simulation involves analyzing several different situations based on an initial model to predict wear.

## Conclusion

A computational simulation is performed to evaluate the wear of four different fixed-bearing patient-specific TKAs with different conformities. The results imply that articular surface conformity in patient-specific TKAs is important. Therefore, our results suggest that it is important to carefully determine conformity when a PE insert is designed in patient-specific TKAs.

## Data Availability

Not applicable

## Abbreviations

3D: Three-dimensional
AP: Anterior-posterior
BPS-TKA: Bio-mimetic patient-specific TKA with a patient’s own tibial and femoral anatomy
CAD: Computer-aided design
CPS-TKA: Conventional Genesis II TKA, patient-specific TKA with Genesis II conformity
CR: Cruciate retaining
FE: Finite element
IE: Internal-external
IS: Inferior-superior
ML: Medial-lateral
MPS-TKA: Patient-specific TKA with medial pivot conformity
PE: Polyethylene
TKA: Total knee arthroplasty
VV: Varus-valgus

## References

[1] Franklin PD, Karbassi JA, Li W (2010). Reduction in narcotic use after primary total knee arthroplasty and association with patient pain relief and satisfaction. J Arthroplasty.

[2] Khatri PJ, O'Connor AM, Dervin GF (2011). Decision support needs of patients choosing between unicompartmental and total knee arthroplasty for advanced medial compartment osteoarthritis of the knee. J Arthroplasty.

[3] Lovald ST, Ong KL, Lau EC (2013). Mortality, cost, and health outcomes of total knee arthroplasty in Medicare patients. J Arthroplasty.

[4] Choi YJ, Lee KW, Kim CH (2012). Long-term results of hybrid total knee arthroplasty: minimum 10-years follow-up. Knee Surg Relat Res.

[5] Meftah M, Ranawat AS, Ranawat CS (2012). Ten-year follow-up of a rotating-platform, posterior-stabilized total knee arthroplasty. J Bone Joint Surg Am.

[6] Parvizi J, Nunley RM, Berend KR (2014). High level of residual symptoms in young patients after total knee arthroplasty. Clin Orthop Relat Res.

[7] Von Keudell A, Sodha S, Collins J (2014). Patient satisfaction after primary total and unicompartmental knee arthroplasty: an age-dependent analysis. Knee.

[8] Devers BN, Conditt MA, Jamieson ML (2011). Does greater knee flexion increase patient function and satisfaction after total knee arthroplasty?. J Arthroplasty.

[9] Noble PC, Gordon MJ, Weiss JM, et al. Does total knee replacement restore normal knee function? Clin Orthop Relat Res. 2005:431157–65. 10.1097/01.blo.0000150130.03519.fb 15685070

[10] Parsley BS, Bertolusso R, Harrington M (2010). Influence of gender on age of treatment with TKA and functional outcome. Clin Orthop Relat Res.

[11] Kang KT, Son J, Kwon OR (2016). Morphometry of femoral rotation for total knee prosthesis according to gender in a Korean population using three-dimensional magnetic resonance imaging. Knee.

[12] Kang KT, Son J, Kwon OR (2017). Effects of measurement methods for tibial rotation axis on the morphometry in Korean populations by gender. Knee.

[13] Patil S, Bunn A, Bugbee WD (2015). Patient-specific implants with custom cutting blocks better approximate natural knee kinematics than standard TKA without custom cutting blocks. Knee.

[14] Maniar RN, Singhi T (2014). Patient specific implants: scope for the future. Curr Rev Musculoskelet Med.

[15] Martin GM. In-vivo tibial fit analysis of patient specific TKA system versus off-the-shelf TKA. JISRF Reconstr Rev. 2014;4102. 10.1055/s-0038-1653966 29801179

[16] Schwechter EM, Fitz W (2012). Design rationale for customized TKA: a new idea or revisiting the past?. Curr Rev Musculoskelet Med.

[17] Sharkey PF, Hozack WJ, Rothman RH, et al. Insall Award paper. Why are total knee arthroplasties failing today? Clin Orthop Relat Res. 2002:4047–13. 10.1097/00003086-200211000-00003 12439231

[18] Koh YG, Son J, Kwon OR (2019). Tibiofemoral conformity variation offers changed kinematics and wear performance of customized posterior-stabilized total knee arthroplasty. Knee Surg Sports Traumatol Arthrosc.

[19] Kang KT, Koh YG, Son J (2017). Finite element analysis of the biomechanical effects of three posterolateral corner reconstruction techniques for the knee joint. Arthroscopy.

[20] Kang KT, Koh YG, Son J (2018). Influence of increased posterior tibial slope in total knee arthroplasty on knee joint biomechanics: a computational simulation study. J Arthroplasty.

[21] ConfirMIS. http://www.conformis.com/, Accessed 15 Nov 2019.

[22] Harrysson OL, Hosni YA, Nayfeh JF. Custom-designed orthopedic implants evaluated using finite element analysis of patient-specific computed tomography data: femoral-component case study. BMC Musculoskelet Disord. 2007;891. 10.1186/1471-2474-8-91 PMC2100040 17854508

[23] Karmal R, Kumar A (2013). Three-dimensional (3D) modeling of the knee and designing of custom made knee implant using mimics software. International Journal of Current Engineering and Technology.

[24] Steklov N, Slamin J, Srivastav S, et al. Unicompartmental knee resurfacing: enlarged tibio-femoral contact area and reduced contact stress using novel patient-derived geometries. Open Biomed Eng J. 2010:485–92. 10.2174/1874120701004010085 PMC2866246 20461223

[25] Van Den Heever DJ, Scheffer C, Erasmus P (2011). Contact stresses in a patient-specific unicompartmental knee replacement. Clin Biomech (Bristol, Avon).

[26] Kurtz WB, Slamin JE, Doody SW (2016). Bone preservation in a novel patient specific total knee replacement. Reconstructive Review.

[27] Koh YG, Son J, Kwon SK (2017). Preservation of kinematics with posterior cruciate-, bicruciate- and patient-specific bicruciate-retaining prostheses in total knee arthroplasty by using computational simulation with normal knee model. Bone Joint Res.

[28] Kwon OR, Kang KT, Son J (2014). Biomechanical comparison of fixed- and mobile-bearing for unicomparmental knee arthroplasty using finite element analysis. J Orthop Res.

[29] Kang KT, Park JH, Lee KI (2012). Gait cycle comparions of cruciate sacrifice for total knee design.-explicit finite element. Int J Precis Eng Man.

[30] Kang KT, Son J, Kim HJ (2017). Wear predictions for UHMWPE material with various surface properties used on the femoral component in total knee arthroplasty: a computational simulation study. J Mater Sci Mater Med.

[31] Godest AC, Beaugonin M, Haug E (2002). Simulation of a knee joint replacement during a gait cycle using explicit finite element analysis. J Biomech.

[32] Knight LA, Pal S, Coleman JC (2007). Comparison of long-term numerical and experimental total knee replacement wear during simulated gait loading. J Biomech.

[33] Halloran JP, Easley SK, Petrella AJ (2005). Comparison of deformable and elastic foundation finite element simulations for predicting knee replacement mechanics. J Biomech Eng.

[34] Inoue S, Akagi M, Asada S (2016). The valgus inclination of the tibial component increases the risk of medial tibial condylar fractures in unicompartmental knee arthroplasty. J Arthroplasty.

[35] Pal S, Haider H, Laz PJ (2008). Probabilistic computational modeling of total knee replacement wear. Wear.

[36] Sarkar AD. Friction and wear: Academic Press, Illustrated; 1980.

[37] Abdelgaied A, Liu F, Brockett C (2011). Computational wear prediction of artificial knee joints based on a new wear law and formulation. J Biomech.

[38] McGloughlin TM, Murphy DM, Kavanagh AG (2004). A machine for the preliminary investigation of design features influencing the wear behaviour of knee prostheses. Proc Inst Mech Eng H.

[39] DesJardins JD, Burnikel B, LaBerge M (2008). UHMWPE wear against roughened oxidized zirconium and CoCr femoral knee components during force-controlled simulation. Wear.

[40] Martin G, Swearingen A, Culler S (2014). Hospital outcomes and cost for patients undergoing a customized individually made TKA vs off-the-shelf TKA. JISRF Reconstr Rev.

[41] Varadarajan KM, Zumbrunn T, Rubash HE (2015). Cruciate retaining implant With biomimetic articular surface to reproduce activity dependent kinematics of the normal knee. J Arthroplasty.

[42] Iwaki H, Pinskerova V, Freeman MA (2000). Tibiofemoral movement 1: the shapes and relative movements of the femur and tibia in the unloaded cadaver knee. J Bone Joint Surg Br.

[43] Johal P, Williams A, Wragg P (2005). Tibio-femoral movement in the living knee. A study of weight bearing and non-weight bearing knee kinematics using 'interventional' MRI. J Biomech.

[44] Karrholm J, Brandsson S, Freeman MA (2000). Tibiofemoral movement 4: changes of axial tibial rotation caused by forced rotation at the weight-bearing knee studied by RSA. J Bone Joint Surg Br.

[45] Asano T, Akagi M, Tanaka K (2001). In vivo three-dimensional knee kinematics using a biplanar image-matching technique. Clin Orthop Relat Res.

[46] Dennis DA, Mahfouz MR, Komistek RD (2005). In vivo determination of normal and anterior cruciate ligament-deficient knee kinematics. J Biomech.

[47] Hill PF, Vedi V, Williams A (2000). Tibiofemoral movement 2: the loaded and unloaded living knee studied by MRI. J Bone Joint Surg Br.

[48] Moro-oka TA, Hamai S, Miura H (2007). Can magnetic resonance imaging-derived bone models be used for accurate motion measurement with single-plane three-dimensional shape registration?. J Orthop Res.

[49] Causero A, Di Benedetto P, Beltrame A (2014). Design evolution in total knee replacement: which is the future?. Acta Biomed.

[50] Dennis DA, Komistek RD, Mahfouz MR (2003). Multicenter determination of in vivo kinematics after total knee arthroplasty. Clin Orthop Relat Res.

[51] Massin P, Gournay A (2006). Optimization of the posterior condylar offset, tibial slope, and condylar roll-back in total knee arthroplasty. J Arthroplasty.

[52] Pritchett JW (2004). Patient preferences in knee prostheses. J Bone Joint Surg Br.

[53] Ries MD (2007). Effect of ACL sacrifice, retention, or substitution on kinematics after TKA. Orthopedics.

[54] Schmidt R, Komistek RD, Blaha JD (2003). Fluoroscopic analyses of cruciate-retaining and medial pivot knee implants. Clin Orthop Relat Res.

[55] van Duren BH, Pandit H, Beard DJ (2007). How effective are added constraints in improving TKR kinematics?. J Biomech.

[56] Varadarajan KM, Harry RE, Johnson T (2009). Can in vitro systems capture the characteristic differences between the flexion-extension kinematics of the healthy and TKA knee?. Med Eng Phys.

[57] Abdelgaied A, Brockett CL, Liu F (2014). The effect of insert conformity and material on total knee replacement wear. Proc Inst Mech Eng H.

[58] Brockett CL, Carbone S, Fisher J (2018). Influence of conformity on the wear of total knee replacement: An experimental study. Proc Inst Mech Eng H.

[59] Freeman MA, Pinskerova V (2005). The movement of the normal tibio-femoral joint. J Biomech.

[60] Burton A, Williams S, Brockett CL (2012). In vitro comparison of fixed- and mobile meniscal-bearing unicondylar knee arthroplasties: effect of design, kinematics, and condylar liftoff. J Arthroplasty.

